# State Medical Society of Wisconsin

**Published:** 1888-07

**Authors:** 


### STATE MEDICAL SOCIETY OF WISCONSIN.

### Forty-Second Annual Session, Held in Milwaukee June 6, 1888.

Dr. L. G. Armstrong, of Boscobel, President.

### First Day—Morning Session.

Dr. J. R. Barnett, of Neenah, read a paper on "The Antipyretic and Abortive Treatment of Typhoid and Remittent Fevers." He maintained that the bacillary origin of typhoid fever is so generally conceded that it may be accepted by practical physicians. This affords the basis for a rational search for some antidote to the disease—that is, for some agent inimical to the existence or development of the micro-organism, or elimination of the products of its vital activity and decay, or both. And upon this belief in some infecting microbe as the cause of typhoid fever the specific treatment as it stands to-day has been built.

"Therapeutics of Oxygen" was the title of a paper by Dr. A. J. Hodgson, of Waukesha. He believes that it stimulates the organic activity of the red-blood globules, and can be used in anæmia, albuminuria, all wasting diseases, and dyspepsia. He attributes the sources of failure in its use to, (1) impure gases used; (2) want of proper discretion in the selection of cases; (3) injudicious exhibition; (4) neglect to regulate the personal habits of the patients; (5) neglecting to use rational and necessary auxiliaries; (6) want of due perseverance.

He reported the following case of Bright's disease: The urine showed great profusion of uric acid crystals, and, when boiled, it could not be poured out of the test-tube. By weight there was 3½ per centum of albumen. Countenance was pale, appetite very poor. The patient was given eight gallons of oxygen a day for seven weeks, his condition at the end of that time being as follows: Casts and uric acid had almost entirely disappeared; albumen less than one fourth of one per cent.; countenance much more natural, and he could walk four miles a day, whereas at the beginning of treatment he could not walk more than four blocks without great difficulty. To-day he is in business and feels well.

A report of a case of Calculus Renalis by Dr. R. Zimmerman, of New Cassel, was read by title.

Dr. J. S. Walbridge, of Berlin, read a paper entitled, "Observations on Influenza."

He describes three forms: 1. Those in which the respiratory tract and mucous cavities of the head are principally involved. 2. Those in which the nervous symptoms predominate. 3. Those in which the digestive organs are affected, accompanied by symptoms resembling rheumatism.

The indications for treatment are to relieve distress and support the patent. There are no preventives, no specifics, and expectorants are of but little use. Diaphoretics and anodynes are required in the first stage; quinine, tonics, and nourishment in the latter stages.

### First Day—Afternoon Session.

Dr. Lucy E. Ermine, of Chicago, read a paper on "Electricity as a Practical Therapeutic Agent in Gynæcology," with a report of its use in five cases.

Dr. E. W. Bartlett, of Milwaukee, read a paper entitled "Otitis Media Purulenta," and exhibited and commended the autophone invented and patented by Mr. J. A. Maloney, of Washington, D. C.

FIRST DAY—Evening Session.

Dr. W. P. Hartford, of Beetown, read a paper on "Catarrhal, or Winter Fever."

In the treatment, calomel in grain doses, repeated every four hours, combined with small doses of Dover's powder, has a beneficial effect. Antipyrin gives great relief for several hours, and may have a beneficial action, especially where there is incipient lung complication. In children with congestion of lungs or capillary bronchitis aconite is admissible, but in cases of lobar pneumonia veratrum is better and much easier. On account of the intermittent character of the fever, quinine would seem to be indicated, but it has little or no effect on the temperature; it does not shorten the attack, and often irritates the already weak stomach.

Dr. Townsend read a paper on "The Summer Diarrhœas in Children."

Deaths from intestinal diseases range from 20 to 25 per centum according to location, 85 per centum of these being in children under five years of age.

Treatment must be divided into preventive and medicinal. We should look first to the surroundings, for bad drainage, bad air, overcrowding, dirt, improper food, and clothing.

Professor Leeds, of Stevens Institute, gives the following formula, which he claims as the nearest approach to mother's milk.

1 gill fresh unskimmed cow's milk.
1 gill of water.
2 tablespoonfuls of rich cream.
200 grains of milk sugar.
1¼ grains of extractum pancreatis.
4 grains of bicarbonate of soda.

Put this in a nursing-bottle, and place it into water made so warm that the hand can not be held in it without pain longer than one minute. Keep the contents of the bottle at this temperature twenty minutes, and prepare a fresh lot at each nursing.

SECOND DAY—Morning Session.

Dr. Joseph Schneider, of Milwaukee, read a paper on "Blennorrhœa, Acute and Chronic."

Dr. S. B. Buckmaster, of Mendota, read a paper on "The Importance of a Careful Examination of the Alleged Insane before their Committal to a Hospital."

Almost every person of the present time has some conception of insanity, though that conception is usually of an ideal and exaggerated character.

At the present time there are 3,000 insane persons in the state of Wisconsin.

Of 252 cases admitted to the State hospital in 1887, 38.15 per centum were over 60 years of age. Of 29 deaths during 1887 out of a total of 788 treated, five were between 60 and 70 years of age, and five were over 70.

Dr. J. S. Christison, of Cottage Grove, read a paper entitled "Our Duty to Our Insane."

Dr. James A. Jackson, of Madison, read a paper on "Litholopaxy," with a report of a case.

Dr. G. M. Steele, of Oshkosh, read a paper on "A Clinical Illustration of Localization of Cerebral Function," with a report of a case.

Michael M——, aged 17 years, Norwegian, weight 103 pounds, of slim build, with good family history, except the death of his father from cancer. He was a laborer in a sash, door, and blind factory, and on the morning of July 9, 1887, while cleaning away rubbish from under the table of a machine, he raised his head against a saw of about 9 inches in diameter running at a high rate of speed. The scalp wound extended one-third of an inch to the right of median line, and about one inch posterior to the parieto-frontal articulation over the parietal eminence and downward, describing two curves, and ending about one and three-fourths inches above and a little in front of the left ear. The wound of the parietal bone measured two and three-quarter inches. An estimate of depth of the wound at deepest point made with considerable care placed it at five-eighths of an inch. The hæmorrhage was profuse, yet the boy was brought to the office something more than a mile distant from the factory. His intellect was undisturbed from the reception of the injury, and he complained only of weakness and numbness of his right hand and arm. This condition of the right arm and hand was felt immediately after injury. The wound was carefully cleansed and dressed antiseptically, the upper portion stiched and lower angle left open for drainage. The next day convulsive seizures, slight and of short duration, attacked the affected arm, and the second day after receiving the injury, the whole right side became involved in those convulsive movements, which were not severe. There were three between 2.30 and 10 p. m., and five between 10 p. m. and 12 p. m. At 1 a. m on the third day, one occurred lasting ten minutes, another lasting seven minutes at 1.45 a. m. The wound, which had closed very perfectly, was reopened, and while it was clean and no discharge could be observed to escape, the convulsions ceased and recovery was rapid, perfect, and without complications.

Chloral and the bromides were used quite freely until the unpleasant symptoms ceased. His right arm by this time was completely paralyzed, but there was no paralysis elsewhere. Complete paralysis lasted only about two days, when improvement commenced, and he enjoyed quite complete use of the extremity in three weeks. He was never unconscious except when in convulsions. He sat up more or less every day; his appetite was good, bowels regular, urine very good. During the period of severe convulsions his speech was thickened and slow. He resumed his work some six or seven weeks following the accident.

Dr. G. W. Jenkins, of Kilbourn City, read a paper on "Puerperal Eclampsia."

Whatever the pathology, remote, or exciting causes, the most important subject to be considered is the treatment, which consists of two classes, *preventive* and *curative*, and the curative into *medicinal* and *obstetrical*. It was his uniform practice to bleed patients after having thouroughly considered the case, and ascertained the general condition of plethora, and the dangers to be avoided by a timely and judicious bleeding, diminish directly the amount of blood, relieving blood pressure, and congestion of the whole system. During an experience and practice of over 35 years he had about 20 cases and had never lost one.

After bleeding, if the convulsions continue, he puts patients upon chloroform inhalations, relieve the distended bladder, unload the bowels by means of free irrigation, and proceed to empty the uterus by first gradually dilating the uterine canal until the hand can enter the uterus, turn, and deliver. He does not wait, but empties the uterus as soon as possible. If convulsions continue after the uterus is empty, he used hypodermic injections of morphine, chloral per rectum, hydragogue cathartics, and attempted to unload the kidneys by means of diuretics.

Dr. H. F. Sercombe, of Milwaukee, said that she had had quite an extensive experience in private practice and otherwise. While in the lying-in hospital in Vienna she had occasion to witness a great many cases. The patients were usually brought to the hospital in an eclamptic condition, and on admission the assistant physician would secure a large bath-tub, fill it as full as possible, without letting the water run over, with water as warm as the patient could bear. It made no difference as to how far pregnancy was advanced, whether five, six, seven, or eight months. Patients were immersed in this warm water and kept there for thirty to forty minutes. He would then have them wound in heavy blankets, so as to produce perspiration as freely as possible. If the convulsions continued while the patients were being immersed in the bath, he administered chloroform judiciously, and after taking the patient from the bath he would give an enema of chloral hydrate, twenty to forty grains, made into a solution with mucilage. This was the assistant's general treatment. He did not try to interfere with gestation at any time, except where labor was retarded in its progress. There were very rarely more than about six convulsive attacks after the patient was brought into the hospital, and of the twenty-four cases she saw there were only two deaths

Dr. L. H. Hayman, of Boscobel, said: There was one remedy which was suggested to him by Dr. Miller, of Chicago. One of the most valuable remedies, where there is a nervous condition, with headache, and albumen in the urine, was chlorate of potash, tincture of iron, and morphine, given three or four times a day. He found in many cases in which he had administered this remedy it proved very valuable. Some of the cases which were very troublesome, the patients suffering a great deal of pain, irritation, and pressure in the abdominal wall, were relieved by this treatment.

Dr. J. G. Meachem, sr., of Racine, had practiced medicine forty-five years; during that time he had seen a very large number of cases of eclampsia. He had tried, and seen tried, various forms of treatment, and was satisfied that there was no remedy to be compared with blood-letting. He could not recall a single case that had occurred within his memory that recovered where blood-letting was not resorted to.

Officers for 1889.

President—Dr. J. R. Barnett, of Neenah.

First Vice-President—Dr. G. D. Ladd, of Milwaukee.

Second Vice-President—Dr. H. F. Sercombe, of Milwaukee.

Permanent Secretary—Dr. J. T. Reeve, of Appleton.

Assistant Secretary—Dr. J. R. McDill, of Milwaukee.

Next place of meeting, Milwaukee.

